# STAT3 as a critical target of Sijunzi Decoction in the treatment of gastric cancer: evidence from integrated network pharmacology and experimental validation

**DOI:** 10.3389/fmolb.2025.1683806

**Published:** 2025-11-07

**Authors:** XiaoYu Luo, GuiPing Xie, QianYing Tan, YaoHui Wang, HaiDan Wang, Jing Zhai

**Affiliations:** 1 Department of Surgical Oncology, Jiangsu Province Hospital of Chinese Medicine, Affiliated Hospital of Nanjing University of Chinese Medicine, Nanjing, China; 2 Department of Pathology, Jiangsu Province Hospital of Chinese Medicine, Affiliated Hospital of Nanjing University of Chinese Medicine, Nanjing, China; 3 Department of Pharmacology, Jiangsu Province Hospital of Chinese Medicine, Affiliated Hospital of Nanjing University of Chinese Medicine, Nanjing, China

**Keywords:** Sijunzi decoction, gastric cancer, network pharmacology, tumor immune microenvironment, JAK/STAT pathway, STAT3

## Abstract

**Background:**

Gastric cancer (GC) is an importent cause of global cancer mortality, underscoring the need for therapeutic strategies. Traditional Chinese medicine (TCM), particularly Sijunzi Decoction (SJZD), has demonstrated clinical promise as an adjuvant therapy in oncology by improving survival and reducing chemotherapy toxicity. However, the mechanistic basis of SJZD’s anti-tumor activity, especially concerning its potential immunomodulatory effects within a competent tumor microenvironment, remains poorly elucidated due to the complexity of its components and limitations of previous preclinical models.

**Methods:**

The subcutaneous tumor models were established in inbred 615 mice with MFC cells, and RNA-sequencing (RNA-Seq) was performed on tumor tissues to characterize treatment-associated differentially expressed genes across three groups (model, early Sijunzi Decoction, and synchronization Sijunzi Decoction). Network pharmacology analysis predicted the bioactive compounds and putative targets of Sijunzi Decoction, and constructed a compound-target-disease network to explore potential GC-related pathways. The expression profile of STAT3 in gastric cancer tissues from three groups of mice model was examined through Western blotting assays and immunohistochemistry to determine its role in Gastric cancer and its regulatory relationship.

**Results:**

SJZD could prevent tumor growth. Additionally, the earlier Chinese medicine intervention, the more definiter tumor inhibition. The RNA-Seq revealed an immunomodulatory gene signature, as evidenced by the 13 common DEGs significant enrichment in the JAK-STAT pathway. Network pharmacology identified 156 overlapping targets between SJZD and GC, among which STAT3 was recognized as a critical hub gene. Forthermore, Western blot and IHC analysis confirmed that SJZD had downregulated STAT3 protein expression in tumor tissues.

**Conclusion:**

SJZD had a definitely inhibitive effect against GC in mice by regulation the STAT3 expression in JAK/STAT signaling pathway, providing a mechanistic rationale for the potential clinical translation of SJZD in GC treatment.

## 1 Introduction

Gastric cancer (GC) persists as a leading malignancy worldwide with significant mortality (Fitzmaurice et al., 2015). There were over one million new cases and approximately 783,000 deaths globally, ranking as the fifth most commonly diagnosed malignancy and the third leading cause of cancer-related mortality (Bray et al., 2018; Yang et al., 2018). The principal strategies of GC treatment include gastrectomy with D2 node dissection, radiotherapy, and chemotherapy (Machlowska et al., 2020). Immunotherapy and targeted therapy have been emerged as transformative breakthroughs in the area of oncology recent years (Machlowska et al., 2020; Song et al., 2017). Traditional Chinese medicine (TCM) is increasingly recognized in oncology as a complementary approach with a favorable safety profile (Wang et al., 2018). Clinically, employed as an adjuvant therapy, TCM has been shown to improve outcomes such as reduced durg toxicity, enhanced quality of life, and prolonged survival in non-small cell lung cancer (NSCLC) (Chen et al., 2010), as well as improved efficacy and cost-effectiveness in colon adenocarcinoma (Fei et al., 2018). Nevertheless, despite this clinical evidence, the therapeutic mechanisms of TCM remain incompletely understood due to the complexity of its components. Current research indicates that these mechanisms may involve genetic and epigenetic regulation, cancer stem cell modulation, and tumor microenvironment remodeling (Xiang et al., 2019). Sijunzi Decoction (SJZD) is a classical Traditional Chinese herbal formula with a outstanding clinical application in treating gastrointestinal disorders, including gastric cancer (Ding et al., 2022; Li et al., 2022), colorectal cancer (Shang et al., 2023). SJZD, a classical and representative TCM formulation for treating qi deficiency syndrome in TCMs, comprises *Panax ginseng* C. A. Mey (Radix Ginseng), *Atractylodis Macrocephalae Rhizoma* Koidz. (Largehead Atractylodes Rhizome), *Wolfiporia cocos* (F.A. Wolf) Ryvarden & Gilb. (Indian Buead) and *Glycyrrhiza uralensis* Fisch (Licorice). Pharmacological study (Shang et al., 2023) demonstrated that Sijunzi Decoction can restore chemotherapy-impaired NK-cell and K-cell activity while enhancing granulocyte and monocyte function, as well as bone marrow hematopoiesis. Although the latest study (Ding et al., 2022) showed SJZD exerted anti-tumor effects against GC by suppressing angiogenesis and inducing apoptosis through modulation of the PI3K/AKT pathway, these conclusions cannot fully clarify the mechanism of SJZD action on gastric cancer, because this experiment was based on immune deficient mice (NCG mice), which lack a functional adaptive immune system and thus cannot replicate the complex immunosuppressive dynamics of the human tumor microenvironment. To accurately confirm the molecular mechanism of SJZD in GC treatment, a subcutaneous tumor model was established in immunocompetent inbred 615 mice using MFC cells. The use of this syngeneic model, which possesses a healthy immune system, is essential for capturing the full therapeutic profile of SJZD, particularly its critical immunomodulatory effects, thus offering insights with greater clinical translatability. We randomized the MFC tumor-bearing mice into groups based on SJZD treatment and administration schedule. Tumor tissues were subjected to RNA-Seq to compare the gene profiles among these groups. In parallel, we employed network pharmacology to identify the bioactive compounds and putative targets of SJZD. The integration of these two approaches aimed to elucidate the potential mechanisms of SJZD in gastric cancer treatment.

## 2 Materials and methods

### 2.1 Herbal materials and the preparation of SJZD

The herbal components of SJZD were sourced from Jiangsu Province Hospital of Chinese Medicine. This formulation is composed of four herbs, including *P. ginseng* C. A. Mey (Radix Ginseng), *Atractylodis Macrocephalae Rhizoma* Koidz. (Largehead Atractylodes Rhizome), *Wolfiporia cocos* (F.A. Wolf) Ryvarden & Gilb. (Indian Buead) and *G. uralensis* Fisch (Licorice) (Table 1). Crude drug authentication, including both microscopic and macroscopic examination, was conducted by the Jiangsu Province Hospital of Chinese Medicine to verify compliance with the quality standards of the Pharmacopoeia of the People’s Republic of China (Edition 2015). Voucher specimens were deposited in the Institute of Pharmacology laboratory, Jiangsu Province Hospital of Chinese Medicine. The Sijunzi decoction was prepared in the Institute of Pharmacy Department of Jiangsu Province Hospital of Chinese Medicine.

**TABLE 1:** The composition of Sijunzi decoction.

Herbal medicine	Chinese nane	The used park	Quantity (g)	Occupied percent (%)
Radix Ginseng	Renshen	Root	9	27
Largehead Atractylodes Rhizome	Baizhu	Root	9	27
Indian Buead	Fuling	Flesh	9	27
Licorice	Gancao	Root	6	19

In brief, Radix Ginseng (28.8 g) was pretreated with vinegar immersion for 2 h. The rest of the herbs (Largehead Atractylodes Rhizome28.8 g, Indian Buead 28.8 g, and Licorice 19.2 g) underwent aqueous soaking for 1 h. The final combined herbs were decocted in distilled water for 2 h followed by a second extraction (1 h). The filtrate was concentrated to 200 mL by rotating the evaporator, yielding a final concentration of 1.92 g/mL. Then, the solution was diluted with sterile water to achieve the final concentration of 0.96 g/mL.

### 2.2 MFC cell line culture

The mouse GC cell lines MFC (X-Y Biotechnology, Shanghai, China) were cultured in RPMI 1640 supplemented with 10% FBS and 100 U/mL penicillin and streptomycin, until the logarithmic growth phase, and adjust the cell density to 1 × 10^7^/mL.

### 2.3 Establishment of the MFC mice cell line-derived subcutaneous tumor model

**FIGURE 1:**
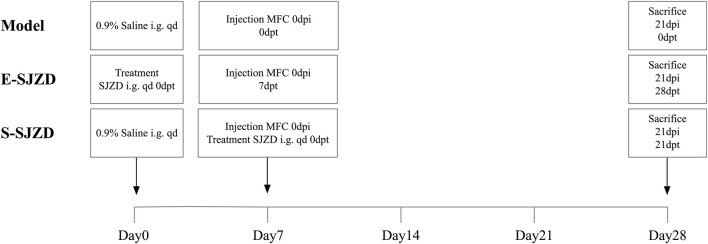
Schematic of the experimental timeline for each group of MFC-bearing 615 mice. i.g., intragastric administration (oral gavage); qd, quaque die (once daily); dpi, days post inoculation; dpt, days post treatment.

The inbred 615 mice (4–5 weeks age), which having the healthy immune system, were obtained from Shanghai Bikai Keyi Biotechnology Co., Ltd. (Shanghai Laboratory Animal Quality Certification, No. 0059551) and maintained under specific pathogen free (SPF) room set at 22 °C ± 2 °C, 55% ± 5% humidity, and a 12/12 circadian rhythm, with *ad libitum* access to water and diet. 0.2 mL MFC cell suspension inoculated with each 615 mice, after disinfecting the right armpit of the mouse with 75% ethanol, dry it with sterilized cotton balls. Following 1 week acclimatization feeding, mice were randomly divided into three experimental groups based on whether to take oral gavage with SJZD and the different time node of Chinese medicine oral gavage. The groups were designed as follows: Model group (model): 200 μL 0.9% saline intragastric administration at first day + injection MFC at seventh day; early Sijunzi Decoction (E-SJZD): 200 μL 19,200 mg/kg/bw Chinese Medicine intragastric administration at first day + injection MFC at seventh day; synchronization Sijunzi Decoction (S-SJZD): both 200 μL 19,200 mg/kg/bw Chinese Medicine intragastric administration and injection MFC at seventh day. Each experimental group consisted of six mice, and mice received intragastric administration daily for 3 consecutive weeks. All rats were sacrificed on 21st day. The experimental timeline and group design were schematically illustrated in Figure 1. All experiments were conducted at the Laboratory Animal Center of Jiangsu Province Hospital of Chinese Medicine in compliance with institutional ethical guidelines (Ethical approval number, 2022-DW-58-02). All experimental procedures were conducted in strict accordance with the National Institutes of Health Guide for the Care and Use of Laboratory Animals and were performed by certified personnel under veterinary supervision.

### 2.4 Determination of tumor size and sample collection

To monitor systemic toxicity and tumor growth, mouse body weight and tumor dimensions were recorded every day. The tumor volume was calculated according to the formula: volume = length × width^2^/2. After the intervention was completed, the tumor had been completely removed after the mice were euthanize.

### 2.5 RNA preparation and sequencing of tumors from MFC mice

RNA from snap-frozen tissues samples was extracted using TRIzol (Invitrogen, United States) according to manufacturer’s instructions. mRNA was isolated from total RNA extracts. Strand-specific RNA-seq library was prepared and subjected to high-throughput sequencing on the DNBSEQ platform. Raw sequencing data were quality-filtered with SOAPnuke (Li et al., 2008). Subsequent analysis and data mining were performed through Dr. Tom Multi-omics Data mining system (https://biosys.bgi.com). Gene expression levels were quantified using RSEM (v1.3.1) (Li and Dewey, 2011). Heatmap visualization of differential gene expression patterns across samples was drawn by pheatmap (v1.0.8). Differential expression analysis was conducted with DESeq2 (v1.4.5) (Love et al., 2014) (or DEGseq (Wang et al., 2010) or PoissonDis (Audic and Claverie, 1997)), applying a significance threshold of Q value ≤ 0.05 (or FDR≤0.001). GO (http://www.geneontology.org/) and KEGG (https://www.kegg.jp/) enrichment analysis of annotated DEGs was performed using the Hypergeometric test in Phyper (https://en.wikipedia.org/wiki/Hypergeometric_distribution). The significance of terms and pathways was assessed with a stringent Q-value threshold (Q ≤ 0.05) for multiple testing correction.

### 2.6 Network pharmacological analysis

**FIGURE 2:**
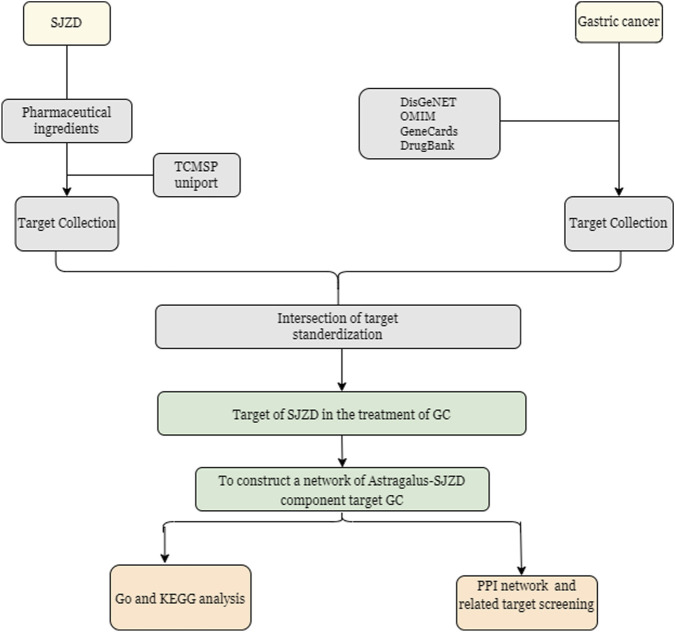
Illustration of Network pharmacology.

The bioactive compounds of SJZD were retrieved from the Traditional Chinese Medicine Systems Pharmacology Database and Analysis Platform (TCMSP, https://old.tcmsp-e.com/tcmsp.php) (Ru et al., 2014) according to commonly used pharmacokinetic screening parameters in network pharmacology: drug likeness (DL) ≥ 0.18 and oral bioavailability (OB) ≥ 30%. Corresponding protein targets of these compounds were then retrieved from TCMSP and standardized to official gene symbols of *Homo sapiens* using the UniProt Knowledgebase (UniProtKB, https://www.uniprot.org/) (UniProt Consortium, 2019). GC-related genes were collected from four public databases with the keyword “gastric cancer”, including GeneCards (https://www.genecards.org/) (Safran et al., 2010), Online Mendelian Inheritance in Man (OMIM, https://www.omim.org/) (Amberger et al., 2015), DrugBank (https://go.drugbank.com/) (Wishart et al., 2018), and DisgeNET (https://www.disgenet.org/) (Pinero et al., 2015). Through Venny (v2.1.0) (http://www.liuxiaoyuyuan.cn/), the overlapping genes between SJZD and GC were searched, and their intersection was visualized as a Venn diagram. A Protein-Protein Interaction (PPI) network for the overlapping genes was constructed utilizing the STRING database (Search Tool for Retrieval of Interacting Genes/Proteins) (http://string-db.org/) (Szklarczyk et al., 2021) with a confidence score ≥0.7 to ensure biological relevance. The resulting network was imported to Cytoscape software (v3.9.1) (Doncheva et al., 2019) for visualization and further analysis. In the network, nodes represent proteins while edges represent functional associations, with edge thickness indicating the confidence level of the intersection. Only connected nodes were retained. Subsequently, hub genes were selected based on high-degree node centrality calculated by Metascape (https://www.metascape.org) (Zhou et al., 2019) in order to analyze the biological functions and pathways, involving Gene Ontology (GO) and Kyoto Encyclopedia of Genes and Genomes (KEGG) enrichment. GO categorization included three domains: (1) cellular component (CC), (2) molecular function (MF), and (3) biological process (BP) (Ashburner et al., 2000). KEGG pathway analysis provided systematic functional gene annotation. The analysis was performed with the following parameters: minimum overlap = 3, minimum enrichment = 1.5, and an adjusted p-value below 0.01. Results were visualized as bubble charts using the bioinformatics online platform (http://www.bioinformatics.com.cn/). The overall experimental design and analytical workflow are schematically illustrated in Figure 2.

### 2.7 Western blot

Following protein extraction, concentrations were determined using a bicinchoninic acid (BCA) assay kit (Beyotime Biotechnology, China). Samples were then separated by sodium dodecyl sulfate-polyacrylamide gel electrophoresis (SDS-PAGE), transferred onto PVDF membranes, and blocked with 5% non-fat milk for 1 h at room temperature. The PVDF membrane was first incubated overnight at 4 °C with primary antibody solution (anti-STAT3, clone 124H6; Cell signaling, United States, 1:1,000 dilution). Subsequently, the membrane was incubated with HRP-conjugated goat anti-mouse secondary antibody (1:1,500 dilution; A0216, Beyotime Institute of Biotechnology, Shanghai, China) for 1 h at room temperature. Finally, protein signals were detected using the enhanced chemiluminescence (ECL) reagents (Beyotime Biotechnology, China) under dark conditions and imaged with chemiluminescence detection system.

### 2.8 Immunohistochemistry (IHC) staining

Tissue samples were fixed, paraffin-embedded, and processed for IHC staining according to standard protocols. After deparaffinization and antigen retrieval, tissue sections were washed three times with PBS and then incubated overnight at 4 °C with STAT3 (1:200 dilution) primary antibody. Subsequently, the slices were incubated with the corresponding secondary antibody and were counterstained with hematoxylin before mounting. STAT3 immunopositivity was quantitatively analyzed by ImageJ software. The protein expression score was determined by multiplying the percentage of positive area (quantified by ImageJ) by the staining intensity (graded as: 1 = mild, 2 = moderate, 3 = strong).

### 2.9 Statistical analysis

Data are expressed as mean ± standard error of the mean (SEM). Statistical significance was estimated using Analysis of Variance (ANOVA). According to the result of homogeneity of variance test, the data statistical analysis was conducted using LSD (Least-Significant Difference) or Tamhane T2. Protein or gene expression levels were determined in at least three independent experimental replicates. Quantitative comparisons between control and treatment groups were performed by analysis of variance. All analyses were conducted with SPSS software (version 22.0; SPSS Inc., Chicago, IL). Statistical significance was defined as two-tailed P-value <0.05 for all tests.

## 3 Result

### 3.1 Sijunzi Decoction repressed the tumor growth and body mass of MFC mice

**FIGURE 3:**
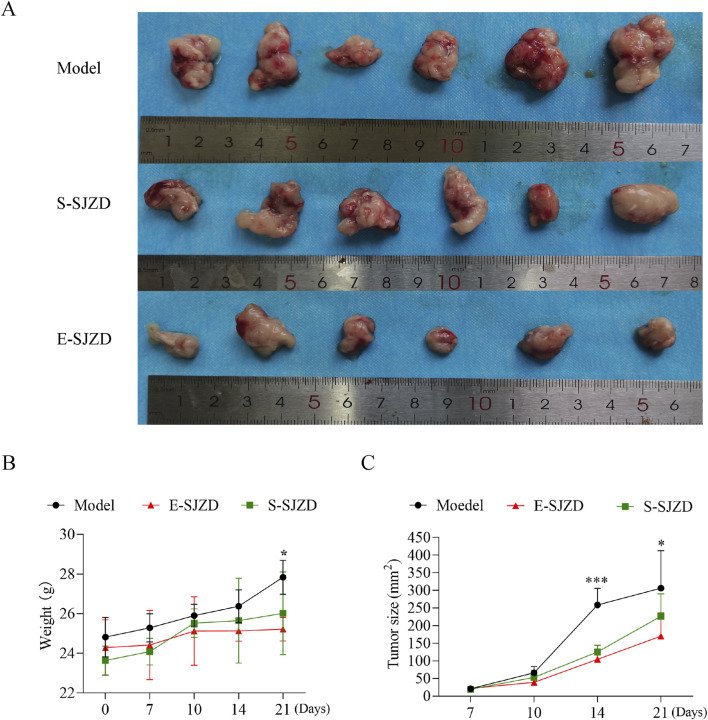
SJZD suppressed tomorigenesis in 615 mice of MFC. **(A)** Photograph of tumor excised from the three groups of 615 mice models. **(B)** Body mass of mice after SJZD treatment. **(C)** Tumor volume measurements following SJZD treatment. Data represent as the mean ± SD of three independent experiments, *P < 0.05; ***P < 0.001.

Compared with the Model group, a statistically significant difference in body mass was observed on the 21st day (Figure 3B *P* = 0.013) and the *post hoc* comparison analysis showed the remarkable difference between E-SJZD and Model group (*P* = 0.001). During tumor growth monitoring, we found that the tumor size in the mice of E-SJZD and S-SJZD was both significantly reduced on the 14th day compared with Model group (*P* < 0.0001), however, on the 21st day, there were only noticeable difference between the E-SJZD and Model group (*P* = 0.011, Figures 3A‐C). These data indicated that SJZD could prevent tumor growth, and that the earlier Chinese medicine intervention was administered, the more sustainable tumor inhibition was.

### 3.2 Comprehensive analysis of mRNA expression signature in GC cancer tissue of MFC mice

**FIGURE 4:**
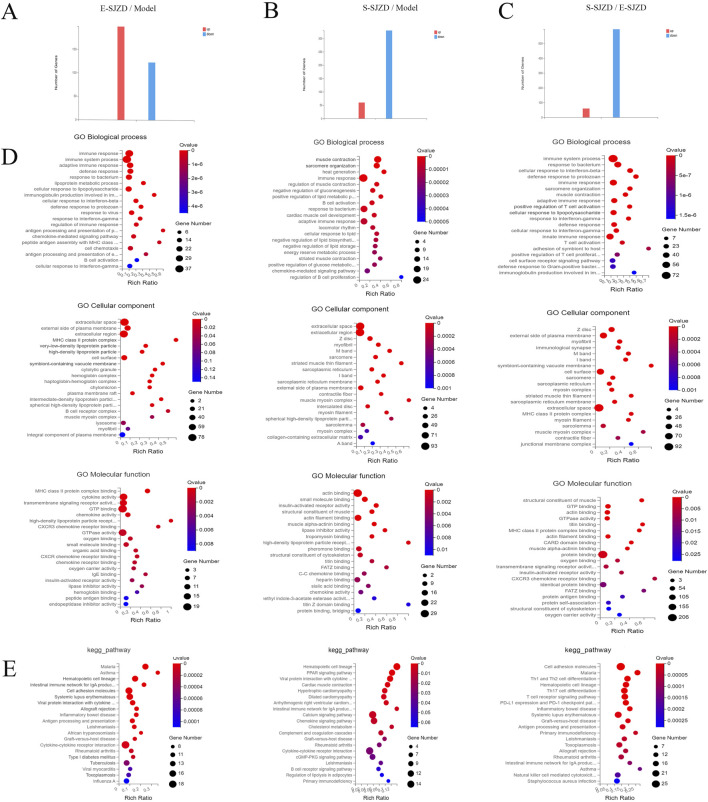
Comprehensive analysis mRNA expression signature in GC cancer tissue among three groups of MFC mice. **(A–C)** The DEGs of mRNA expression among three groups. **(D)** GO functional enrichment analysis. The top 20 terms from each GO category (BP, CC, and MF) were selected. **(E)** Analysis of KEGG pathway enrichment.

In order to identify effects of SJZD on tumor growth in MFC mice, we collected the GC tumor in the three groups, and performed the RNA sequencing. This was done to compare the gene profiles of MFC mice with or without SJZD, and the different Chinese medication durations (E-SJZD and S-SJZD). Transcriptomic analysis revealed 322 differentially expressed genes (DEGs) between E-SJZD and Model groups, comprising 199 upregulated and 123 downregulated genes (Figure 4A). Comparison of the S-SJZD and Model groups revealed 390 DEGs, containing 60 upregulated and 330 downregulated (Figure 4B). A total of 657 DEGs were identified in the S-SJZD group compared to the E-SJZD group, comprising 598 genes downregulated and 59 genes upregulated (Figure 4C). Based on the Metascape database, the top 20 significantly enriched clusters in BPs, CCs, and MFs were selected, and GO enrichment analysis was performed on them (Figure 4D). Additionally, we identified the top 20 enriched KEGG pathways (Figure 4E). We focused on the DEGs among the Chinese medicine groups and Model group. GO enrichment analysis demonstrated the DEGs were mainly enriched in “Immune response”, “extracellular space” and “chemokine activity”. Subsequently, KEGG pathway analysis revealed predominant involvement in the cytokine and chemokine interaction pathways, including “cytokine-cytokine receptor interaction”, “chemokine signal pathway”. Together, these data indicated that these DEGs were primarily implicated in immune function regulated by cytokines and chemokines.

**FIGURE 5:**
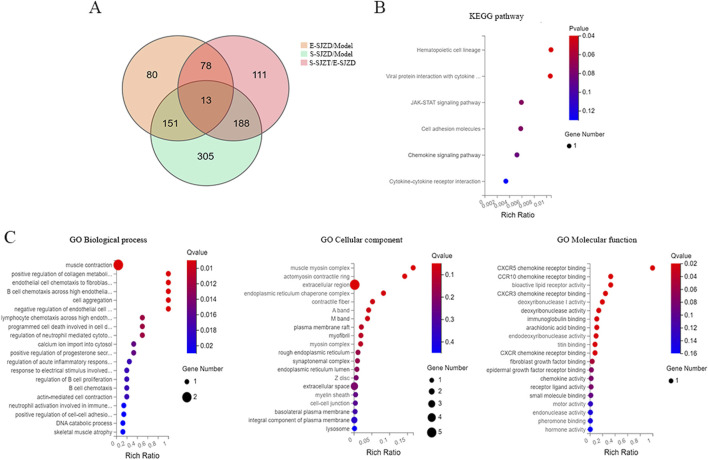
Comprehensive analysis of common targets DEGs in GC cancer tissue of MFC mice. **(A)** Venn diagram of DEGs among three groups. **(B)** Analysis of KEGG pathway enrichment of 13 common DEGs. **(C)** GO functional enrichment analysis of 13 common DEGs.

After intersecting these DEGs among three groups, we obtain 13 common targets DEGs (Figure 5A). Six main pathways with these common targets DEGs were enriched by KEGG analysis, including hematopoietic cell lineage, viral protein interaction with cytokine, JAK-STAT signaling pathway, cell adhesion molecules, chemokine signaling pathway, cytokine-cytokine receptor interaction (Figure 5B). Subsequently, the GO term enrichment analysis of those genes, which revealed that these DEGs were predominantly enriched within the muscle contraction, extracellular region and cytokine receptor binding (Figure 5C).

### 3.3 Network pharmacology-based investigation of Sijunzi Decoction’s therapeutic mechanism

**FIGURE 6:**
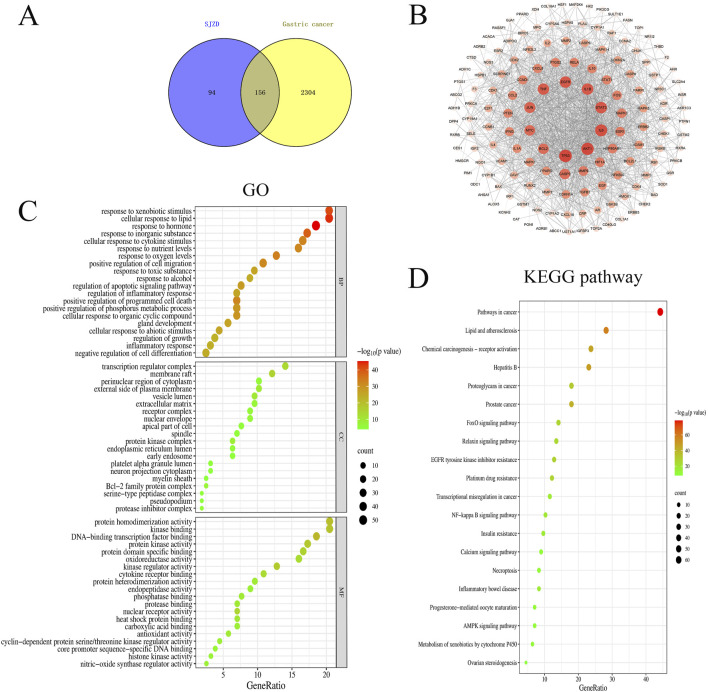
Network of the main pathways and targets of Sijunzi Decoction treatment of Gastric cancer. **(A)** Venn diagram of active ingredients and disease targets. **(B)** PPI network. The darker the color of the circle is, the larger the shape and the higher the degree value of the target. Gene ontology GOfunctional **(C)** and KEGG pathway enrichment analyses **(D)**.

To elucidate the therapeutic mechanism of SJZD in GC, a network pharmacology approach was employed. Bioactive compounds of SJZD were retrieved from the TCMSP database, yielding 22 compounds from *Radix Ginseng*, 16 compounds from *Atractylodes Macrocephalae*, 15 compounds from *Wolfporia cocos*, and 92 compounds from *G. uralensis*. Target prediction analysis identified 114, 17, 23, and 230 potential targets for these herb-derived compounds, respectively. After merging and removing duplicates, 250 unique protein targets were retained as potential SJZD targets. From comprehensive database mining (DisGeNET, OMIM, GeneCards and DrugBank), 3,449 GC-related genes were initially collected. After deduplication, 2,459 GC-related genes were retained. Intersection analysis between SJZD targets and GC genes revealed 156 overlapping targets (Figure 6A), which were considered putative therapeutic targets of for further analysis. A PPI network was constructed from these 156 overlapping targets through the STRING database, with a significance threshold of PPI enrichment *p* < 0.01 (Figure 6B). The top 10 hub genes were identified based on node degree (largest node size and deepest color in the network), with TP53, AKT1, IL6, STAT3, IL1B, EGFR, TNF, JUN, MYC and BCL2 all exhibiting degree values greater than 90. This suggested that these targets represent core regulatory nodes with high biological relevance to GC pathogenesis and potential therapeutic value for SJZD. GO enrichment analysis revealed significant involvement of the target genes in responses to hormones, transcription regulator complex and DNA-binding transcription factor binding (Figure 6C). Complementary KEGG analysis further corroborated the role of SJZD in Pathways in cancer (Figure 6D).

### 3.4 SJZD treatment GC via reducing STAT3 via JAK-STAT pathway

**FIGURE 7:**
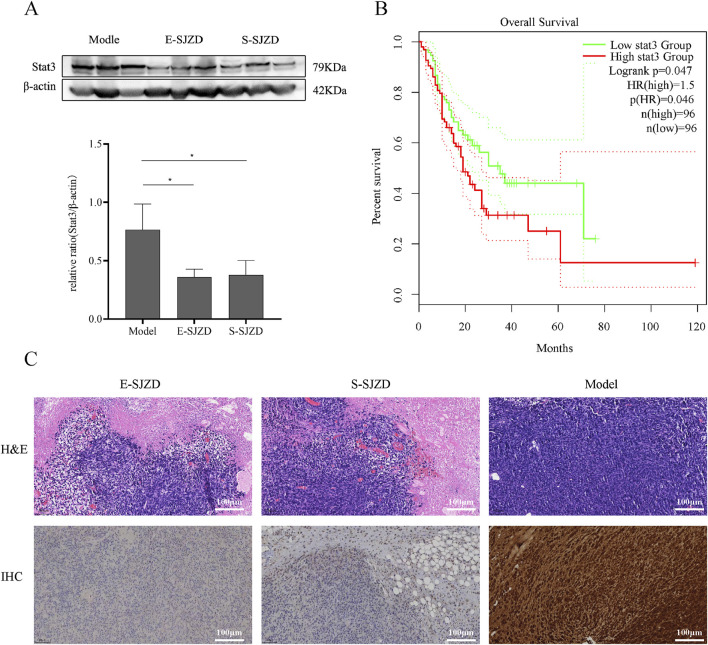
SJZD can inhibit tumor proliferation by regulation the STAT3 expresstion levels. **(A)** Representative images of Western blot. Image gel was used to quantify the expression of STAT3. **(B)** GEPIA database analysis of the relationship between STAT3 expression leves and the Percent survival in GC patients. **(C)** The tumors were stained with H&E and IHC (STAT3). Data are expressed as the means ± SD. Compared with the model group, *P < 0.05.

Due to the Network Pharmacology Analysis on SJZD and the data analysis of 13 common targets DEGs, the therapeutic mechanism of SJZD against GC was shown to associate with STAT3 via JAK-STAT pathway. We investigated STAT3 expression status across three distinct GC mice models tissues. Western blotting assays showed the STAT3 expression levels in gastric cancer tissues of mice model group was much higher compared to other two groups (Figure 7A). Next, GEPIA database (http://gepia.cancer-pku.cn/) was used for survivals analysis which revealed significantly more dismal prognosis in GC patients with high STAT3 expression compared to those with low expression (n = 96) (*P* = 0.046) (Figure 7B). Besides, immunohistochemistry (IHC) staining showed that the number of STAT3-positive tumor cells in the model group was significantly higher than that in the other two TCM intervention groups (Figure 7C).

## 4 Discussion

While TCMs have demonstrated promising anti-tumor effects in GC across multiple published clinical studies (Lu et al., 2022; Xu et al., 2022), the insufficient mechanistic elucidation has hindered their widespread clinical adoption. Our previous clinical studies have shown the effect of SJZD against GC (Xie et al., 2022). In the present study, SJZD effectively inhibited the tumor proliferation in the MFC mice and earlier intervention resulted in stronger inhibition (Tan et al., 2023). The mechanism of tumor proliferation in GC was attributable to a diverse range of determinants, such as environmental factors, microbiome composition, and the host immune system (Xiang et al., 2019). TCMs exert significant effects on preventing tumor growth and relapse through regulating tumor immune microenvironment, impacting tumor suppressor genes or oncogenes and among other mechanisms (Zhang et al., 2021).

The anti-tumor efficacy of SJZD was confirmed in animal models, showing a clear time-dependent effect. First, both SJZD treatment groups showed significant tumor growth inhibition compared to the Model group. Second, within the treatment groups, the E-SJZD regimen showed a stronger inhibitory effect than the S-SJZD regimen. Collectively, these results highlight the enhanced therapeutic benefit of earlier SJZD oral administration for tumor control, suggesting that the timing of treatment initiation is critical to its efficacy.

Based on the impactful treatment of SJZD against GC observed in MFC mouse model, we performed RNA-Seq on tumor tissues to identify potential therapeutic targets. Intersection of the DEGs across the experimental groups yielded 13 common target genes and implicated 6 major pathways. GO and KEGG enrichment analysis indicated that immunoregulation via the JAK-STAT signaling pathway is a primary mechanism of SJZD. In parallel, network pharmacology analysis identified the top 10 hub genes, which included TP53, AKT1, IL6, STAT3, IL1B, EGFR, TNF, JUN, MYC, and BCL2, highlighting their central roles in GC pathogenesis and potential as therapeutic targets. The convergence of evidence from both RNA-Seq and network pharmacology analysis firmly establishes STAT3 as a key target mediating the anti-GC effects of SJZD. By mechanistically linking the observed *in vivo* efficacy to STAT3 pathway modulation, our work provides a crucial mechanistic foundation for advancing SJZD as a promising therapeutic strategy.

STAT3, a cytoplasmic transcription factor belonging to the Janus kinase (JAK)-signal transducer and activator of transcription (STAT) signaling pathway, seeves as a pivotal regulator of tumorigenesis acting in cancer cells themselves and in immune cells and CAFs within the TME. Numerous recent studies have reported that STAT3 signaling pathway has been extensively validated as a potential therapeutic target in oncology. Furthermore, accumulating evidence underscores constitutive STAT3 activation in orchestrating tumor-mediated immunosuppression through multifaceted mechanisms. Yuan K et al., confirmed that activation of the JAK2/STAT3 signaling pathway promotes disease progression (Yuan et al., 2020). Yuan-Ming Pan et al., indicated that STAT3 activation promotes EZH2 upregulation, which correlates with advanced TNM stage and poor prognosis in GC, suggesting STAT3 inhibition as a novel therapeutic strategy (Pan et al., 2016). Furthermore, activating the JAK2/STAT3 pathway could induce programmed death-ligand 1 (PD-L1) expression, indicating that downregulating STAT3 could activate tumor immunity by inhibiting PD-L1 expression (Wang et al., 2017). Several previous studies also indicated that STAT3 might represent a novel therapeutic target in gastric cancer.

Undoubtedly, the evaluation of the therapeutic effects and the benefits of SJZD for patients with gastric cancer is a highly complex issue. Although our study has confirmed STAT3 as a potential target for SJZD treatment in GC, further experimental validation *in vitro* or *in vivo* is still required to fully elucidate the underlying mechanisms of the effects of SJZD on GC.

## 5 Conclusion

The results of this study established that SJZD had a definitely inhibitive effect against GC in mice by regulation the STAT3 expression in JAK/STAT signaling pathway. Our study also indicated that SJZD maybe a promising treatment for GC via remodeling the tumor immune microenvironment.

## Data Availability

The RNA-seq data presented in this study have been deposited in the ArrayExpress database at EMBL-EBI under accession number E-MTAB-15788. All other supporting data are available in the figshare repository under accession code 10.6084/m9.figshare.30355183.
